# Ribosomal protein S6 kinase 1 promotes the survival of photoreceptors in retinitis pigmentosa

**DOI:** 10.1038/s41419-018-1198-1

**Published:** 2018-11-15

**Authors:** Bin Lin, Guoyin Xiong, Wei Yang

**Affiliations:** 1 School of Optometry, The Hong Kong Polytechnic University, Hung Hom, Kowloon Hong Kong; 2 Department of Ophthalmology, University of Hong Kong, Pokfulam, Hong Kong

## Abstract

Retinitis pigmentosa (RP) is a heterogeneous group of inherited disorders caused by mutations in genes that are mostly expressed by rod photoreceptors, which results in initial death of rods followed by cone photoreceptors. The molecular mechanisms that lead to both rod and cone degeneration are not yet fully understood. The mTOR pathway is implicated in RP. However, it remains unclear whether S6K1 plays an essential role downstream of the mTOR pathway in mediating photoreceptor survival in RP. Our in vitro studies demonstrated that PTEN (phosphatase and tensin homolog) overexpression deactivated mTOR activity and induced 661W cone cell apoptosis. In addition, we identified that S6K1 but not 4EBP1 was the downstream effector of PTEN neurotoxicity using gain- and loss-of-function approaches. Moreover, our in vivo data corroborated the results of our in vitro studies. S6K1 overexpression either in rods or cones promoted these cell survival and function and improved visual performance in the rd10 mouse model of RP. Our data demonstrated that S6K1 was the downstream effector of mTOR and that S6K1 was critical for both rod and cone survival in RP. Our findings make a strong case for targeting S6K1 as a promising therapeutic strategy for promoting the survival of photoreceptors in RP.

## Introduction

Retinitis pigmentosa (RP) is characterized by progressive photoreceptor degeneration and is a leading cause of inherited blindness, affecting 1 in 4000 individuals globally in a diverse group of progressive retinal degenerative diseases^1^. Although in most cases, rod photoreceptors but not cone photoreceptors carry mutated genes, cones die subsequent to the rod cell loss in this disease^2–5^. Since high acuity vision depends primarily on cones, it is the secondary cone death that leads to a reduction in the quality of life for RP patients. However, the molecular mechanisms that trigger both rod and cone degeneration are not fully understood. There is currently no specific treatment for RP.

The mammalian target of rapamycin (mTOR) pathway regulates a signal cascade that promotes cell metabolism, growth, and survival, and mTOR activity is controlled by upstream regulators^6,7^. Aberrant mTOR activation has been implicated in neurological diseases^8^. Similarly, downregulation of the mTOR pathway has been reported in mouse models of RP^9,10^. In addition, several upstream kinases of mTOR are implicated in the regulation of cone cell death. Among these kinases, AKT, a key component of cell survival pathways, is downregulated during the induction of photoreceptor apoptosis in RP retinas^11^. Deletion of one regulatory subunit of the phosphoinositide 3-kinase (PI3K) in cones, a major upstream regulator of AKT, results in cone degeneration^12^. Collectively, these studies point to the importance of maintaining active levels of the mTOR pathway for photoreceptor survival.

The two best-characterized downstream targets of mTOR are ribosomal S6 kinase 1 (S6K1), which phosphorylates ribosomal protein S6, and the eukaryotic initiation factor 4E (eIF4E)-binding protein 4E-BP1. S6K1, a serine/threonine kinase, is phosphorylated and activated by mTOR to promote cell growth, proliferation and differentiation^6^. Previously, S6K1 was reported to play a key role in promoting optic nerve regeneration after crush injury^13^. However, it remains unclear whether S6K1 plays an essential role downstream of the mTOR pathway in mediating both rod and cone cell survival in RP. To address these issues, we initially performed in vitro studies in 661W cone cell line. We found that PTEN overexpression in 661W cone cells resulted in downregulation of the PI3K/mTOR pathway in the 661W cone cells and subsequently induced the 661W cone cell apoptosis. In addition, we identified that S6K1 but not 4EBP1 was the downstream effector of the mTOR pathway in mediating 661W cone cell survival. Moreover, our in vivo data from the rd10 mouse model of RP corroborated the results of our in vitro studies. When we overexpressed S6K1 in rods driven by a bovine rhodopsin promoter (Rho)^14^, we found that S6K1 overexpression improved the rod survival and function in rd10 mice. Similarly, S6K1 overexpression in cones driven by a human red opsin promoter (hRo)^15^ rescued the cone cells from degeneration in rd10 mice. Collectively, our in vitro and in vivo studies confirmed that S6K1 was critical for the survival of both rod and cone cells in the rd10 retina. Therefore, targeting S6K1 might be a potential therapeutic approach for rescuing both rod and cone cells from degeneration in RP.

## Results

### PTEN is upregulated in the rd10 retina

Rd10 mice are a well-characterized mouse model of RP carrying a missense mutation in the beta subunit of rod-specific phosphodiesterase gene 6 (PDE6β) in exon 13, which causes the massive degeneration of rod photoreceptors followed by gradual degeneration of cones^16^. Mutations in this gene have been found in patients with autosomal recessive RP^17^, which makes the rd10 mouse a relevant model for human RP. In rd10 animals, rod cell begins degeneration at postnatal day 18 (P18) and is almost completed by P30^18,19^. Functionally, reduced rod- and cone-dominated electroretinography (ERG) responses are observed in rd10 mice as early as P18 and steadily decline with age^18^. By P30, rod-dominated b-wave response was only 10% while the cone-dominated response was 30% in the rd10 compared to wild-type^18^. In addition, Our group previously reported that cones in rd10 mice had a short outer segment (OS) and a fat inner segment (IS) and reduced cone-dominated ERG responses by P26^20–22^. Similarly, other group also reported the shortening and loss of outer and inner segments of cones by P30 in the rd10^19^. Therefore, we chose P18, P19, and P25 for examining rod cell degeneration, and P25 and P30 for analyzing cone cell degeneration.

**Fig. 1:**
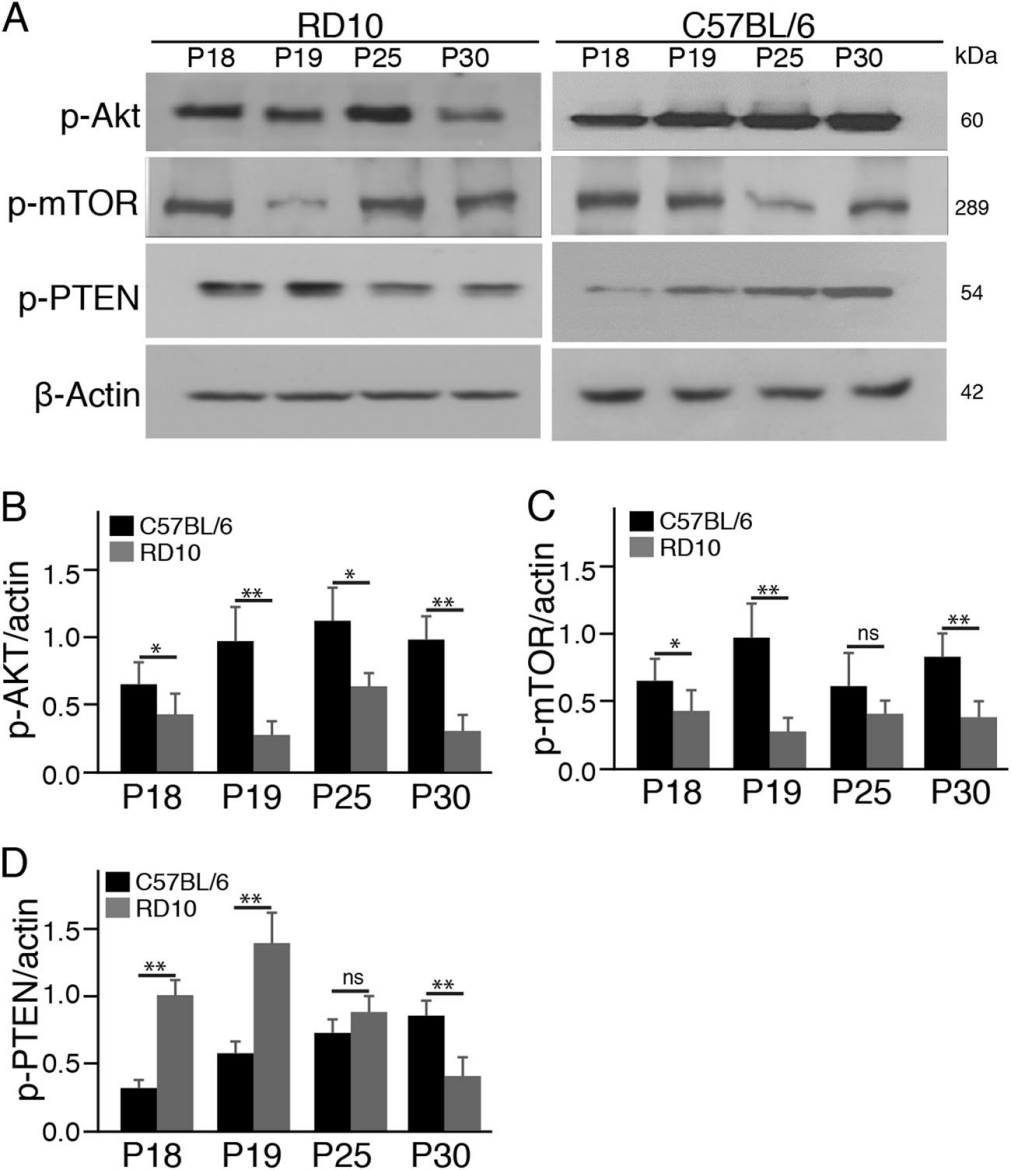
The PI3K/mTOR pathway is downregulated in the rd10 retina. **a** Western blots of whole retinal phosphorylated Akt (p-Akt), p-mTOR, and p-PTEN from rd10 mice and age-matched C57BL/6 mice. β-actin levels were used as a loading control. **b**–**d** Densitometric analyses of the ratio of p-Akt (**b**), p-mTOR (**c**), and p-PTEN (**d**) to β-actin. Data are the mean±SD (*n* = 4). ns, not significant, *p < 0.05, **p < 0.01

The PI3K/Akt pathway, a major pathway mediating neuronal survival, is essential for photoreceptor survival in the mouse retina^11,12^. We initially investigated whether the PI3K/Akt pathway was implicated in photoreceptor degeneration in the rd10 mouse retina. Specifically, we investigated the activation of Akt and mTOR (the mammalian target of rapamycin), two major downstream targets of PI3K. We observed a markedly lower protein level of phosphorylated Akt (p-Akt) in the rd10 retina relative to control C57BL/6J retinas at all these time points (Fig. 1a, b). Similarly, the protein levels of p-mTOR were dramatically decreased in the rd10 retina compared to controls at P18, P19, and P30 (Fig. 1a, c). On the other hand, tumor-suppressor PTEN (phosphatase and tensin homolog), which negatively regulates activity of the PI3K/Akt pathway^6,23^, were found to be dramatically upregulated in the early stage of photoreceptor degeneration in the rd10 retina relative to control C57BL/6J retinas (Fig. 1a, d). Collectively, these findings demonstrated that the PI3K/Akt pathway was implicated in rd10 mice, and suggested a possible correlation between PTEN upregulation and downregulation of the PI3K/Akt pathway in the rd10 retina.

### PTEN overexpression induces 661W cone cell apoptosis in vitro

**Fig. 2:**
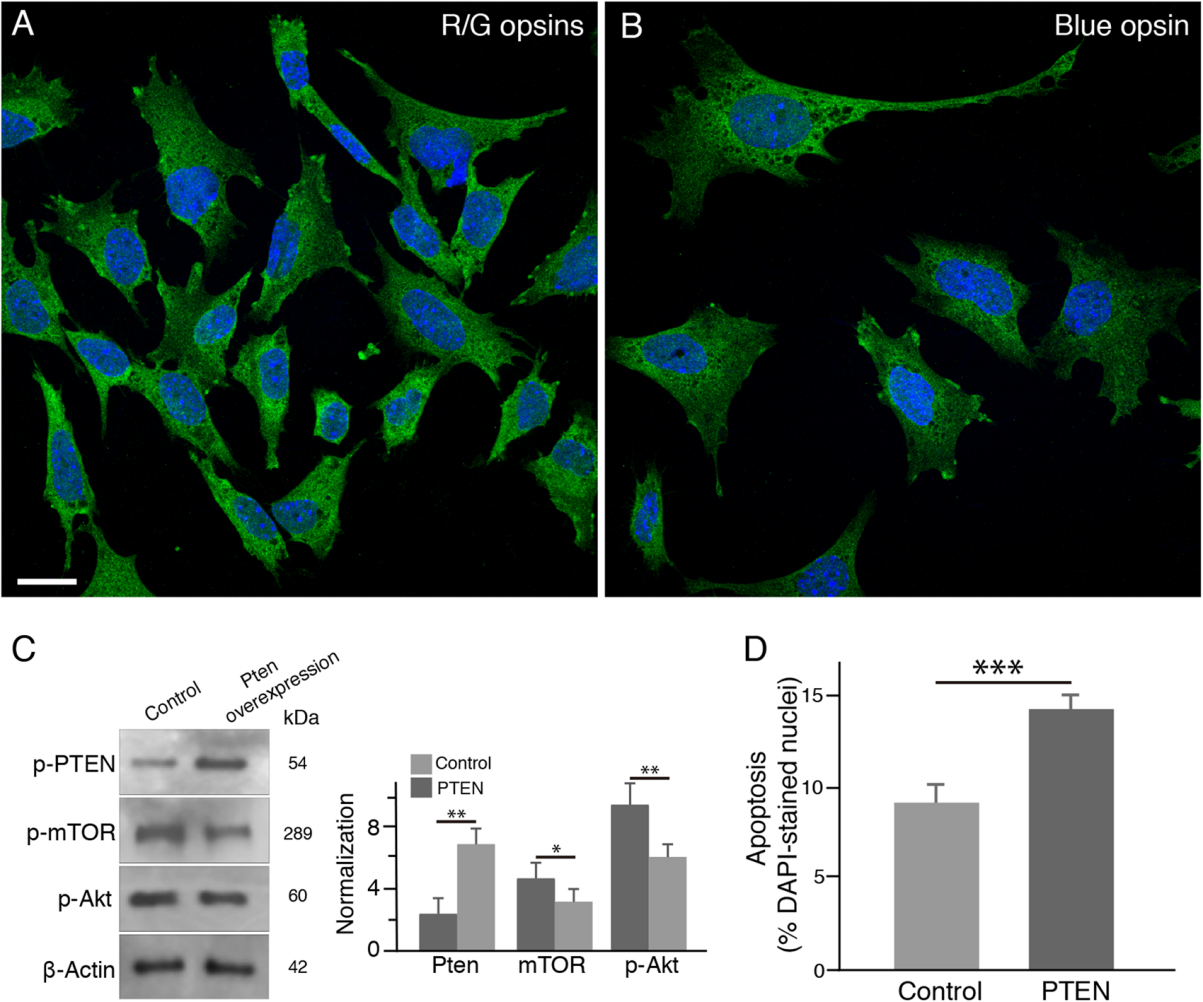
PTEN overexpression downregulates the PI3K/mTOR pathway and induces apoptosis in 661W cone cells in vitro. **a,** **b** 661W cone cells expressed red/green cone opsins (**a**, green) and blue cone opsin (**b**, green). Nuclei were counterstained with the nuclear dye DAPI (blue). Scale bar: 20μm. **c** Western blotting analysis and densitometry of the major downstream targets of the PI3K/mTOR pathway in 661W cone cells after PTEN overexpression. Data are the mean±SD (*n* = 4). PTEN overexpression resulted in downregulation of p-Akt and p-mTOR. *p < 0.05, **p < 0.01. **d** Quantification of TUNEL-positive 661W cells after PTEN overexpression. Results are expressed as the ratio of TUNEL-positive cells to DAPI-stained nuclei. Values represent the mean±SD (*n* = 5). ***p < 0.0001

In the next series of in vitro experiments, we tested whether PTEN overexpression resulted in downregulation of the PI3K/mTOR pathway in 661W cone cell line. The 661W cells, a cone-like transformed cell line^24^, express red/green cone opsins (Fig. 2a) and blue cone opsin as well (Fig. 2b). After PTEN overexpression, we found that p-Akt and p-mTOR expressions were dramatically downregulated in 661W cells (Fig. 2c). To examine whether PTEN overexpression induced 661W cell apoptosis, we stained 661W cells with the terminal deoxynucleotidyl transferase dUTP nick-end labeling (TUNEL). After 24 h in culture, we found that PTEN overexpression significantly increased the proportion of TUNEL-positive 661W cells (Fig. 2d). Taken together, our in vitro results confirmed that PTEN activity downregulated the PI3K/Akt pathway and subsequently induced 661W cell apoptosis in vitro.

### PTEN deletion enhances retinal function and visual performance in rd10 mice in vivo

**Fig. 3:**
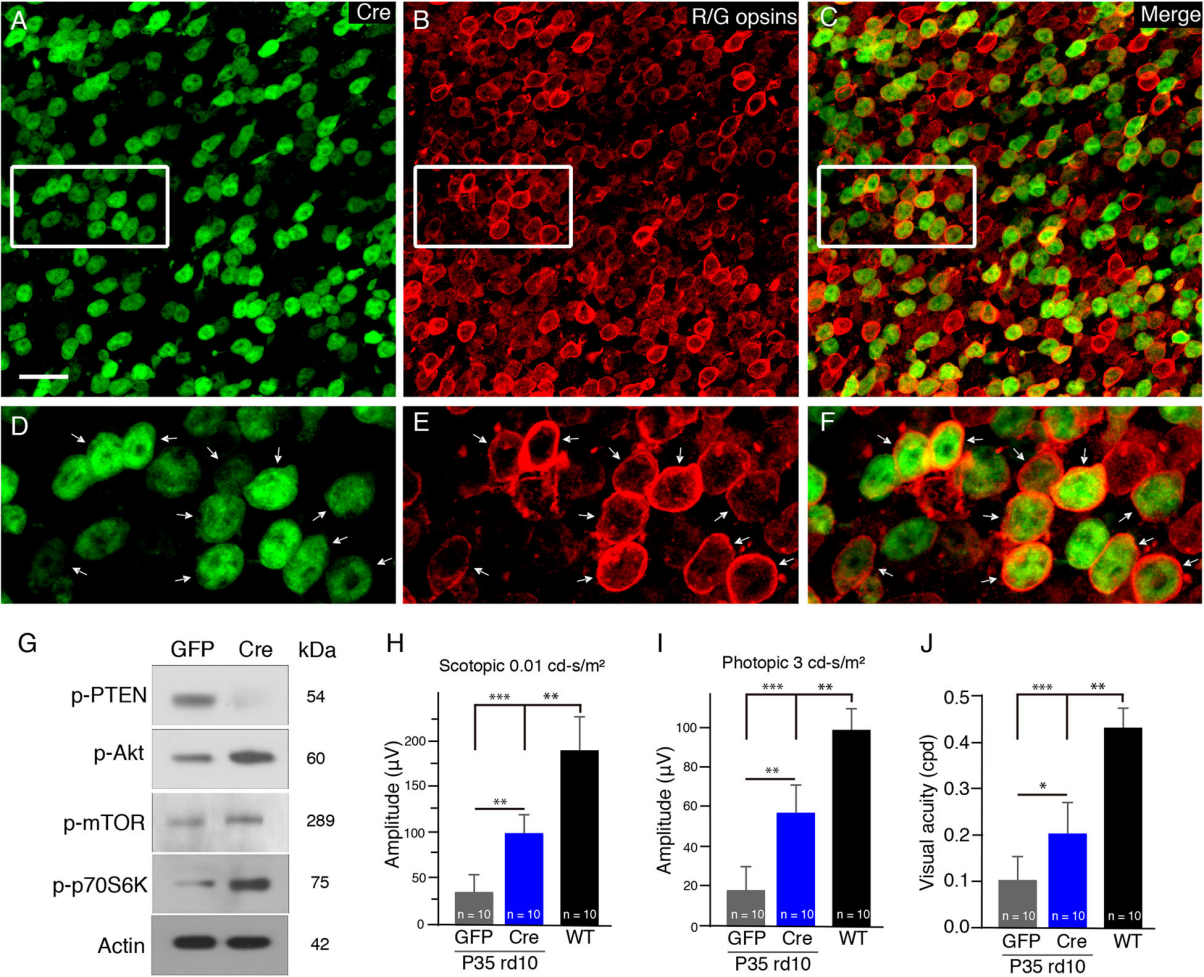
The conditional deletion of PTEN in cone photoreceptors of rd10 mice in vivo. **a–f** Flat-mounted retinas of rd10/PTEN^loxP/loxP^ mice treated with AAV-hRo (human red opsin promoter)-Cre and harvested at P25. Representative retinal flat mounts show extensive Cre expression stained by an anti-Cre antibody (**a**, green) and red/green cones labeled by red/green opsins (**b**, red). The majority of cre-positive cells were also positive for red/green opsins (**c**). **d–f** Illustrate highly magnified images from the boxed regions above, respectively. Arrows indicate the colocalization of Red/green cones with Cre stain. Scale bar: 20μm. **g** Western blots of whole retinal p-PTEN and t-PTEN, p-Akt and t-Akt, p-mTOR and t-mTOR, and p-p70S6K from Cre- or GFP-treated PTEN^loxP/loxP^/rd10 mouse retinas. β-actin levels were used as a loading control. **h** Average scotopic b-wave amplitudes from P35 AAV-Cre- (blue bar) or AAV-GFP-treated PTEN^loxP/loxP^/rd10 mice (gray bar). Age-matched C57BL/6J are shown as comparisons (black bar). **i** Averaged photopic b-wave amplitudes from the three groups of mice. **j** Photopic visual acuity was measured by optokinetic responses. Results are presented as the mean±SD (*n* = 10). *p < 0.05, **p < 0.01, ***p < 0.001

To confirm the involvement of PTEN activity in cone apoptosis in the rd10 retina, we crossed PTEN^loxP/loxP^ mice with rd10 mice and generated a new PTEN^loxP/loxP^/rd10 mouse line, which was homozygous for both the rd10 mutation and the PTEN null allele. To delete PTEN specifically from cones, we injected an adeno-associated virus vector carrying Cre (AAV-Cre) driven by a human red opsin promoter (hRo) into the subretinal space of PTEN^loxP/loxP^/rd10 mice. Assessment of Cre expression and cellular localization in retinas was done by immunostaining using an anti-Cre antibody and an anti-red/green opsin antibody (Fig. 3a–f) and western blot analysis (Fig. 3g). We thus confirmed the conditional deletion of PTEN in cone photoreceptors of rd10 mice.

We then assessed the effect of PTEN deletion on photoreceptor function by measuring scotopic and photopic ERG responses at P35. Scotopic ERG b-wave amplitudes were significantly higher in Cre-treated PTEN^loxP/loxP^/rd10 mice than in GFP-treated PTEN^loxP/loxP^/rd10 mice (5.5-fold; Fig. 3h). Similarly, photopic ERG b-wave amplitudes in Cre-treated PTEN^loxP/loxP^/rd10 mice were larger than that in GFP-treated PTEN^loxP/loxP^/rd10 controls (3.3-fold; Fig. 3i). To evaluate the spatial visual performance in rd10 mice with PTEN deficiency, we measured the optomotor response of mice to moving gratings. Photopic visual acuity was approximately 2.5-fold higher in P35 Cre-treated PTEN^loxP/loxP^/rd10 mice than that in age-matched GFP-treated PTEN^loxP/loxP^/rd10 (*p* < 0.05; Fig. 3j), indicating improved visual acuity in rd10 mice with PTEN deficiency. Consistently, one previous study reported that the conditional deletion of PTEN improved cone survival in rd1 mice^10^. Taken together, we demonstrated that PTEN was involved in the regulation of photoreceptor apoptosis in the rd10 retina, and PTEN deletion improved photoreceptor survival and function in rd10 mice.

### S6K1 is required to maintain the survival of 661W cone cells in vitro

**Fig. 4:**
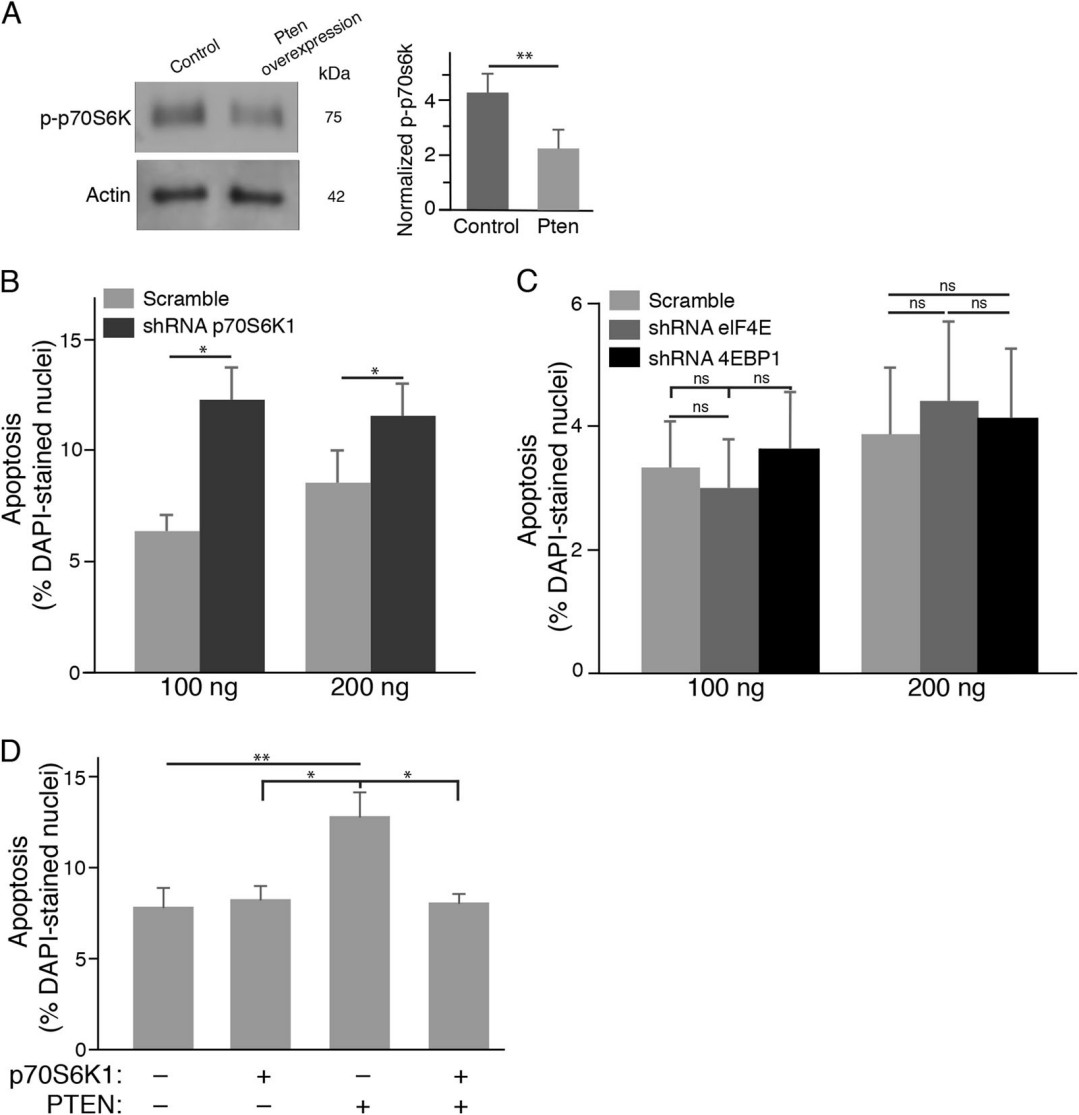
S6K1 is critical for the survival of 661W cone cells in vitro. **a** Western blotting analysis and densitometry of S6K1 protein expressions in 661W cone cells after PTEN overexpression. Results are presented as the mean±SD (*n* = 4). **b** S6K1 knockdown by a specific shRNA against p70S6K1 resulted a higher number of TUNEL-positive 661W cone cells compared with control scramble groups. **c** 661W cone cells were transfected with specific shRNAs against 4EBP1, elF4E, or scramble controls. When compared with scrambled shRNA groups, knockdown of either 4EBP1 or elF4E expression did not affect the survival of 661W cone cells. **d** S6K1 overexpression rescued 661W cone cells in the presence of PTEN. Results are expressed as the ratio of TUNEL-positive cells to DAPI-stained nuclei. Values represent means±SDs (*n* = 4). ns, not significant, *p < 0.05; **p < 0.01

Ribosomal S6 kinase 1 (S6K1) and eukaryotic translation initiation factor 4E-binding protein (4EBP1) are the two main targets of mTOR. After PTEN overexpression in 661W cone cells, we found that S6K1 activity was downregulated (Fig. 4a). To determine the essential role of S6K1 in 661W cone cell survival, we transfected 661W cone cells with p70S6K1 shRNA plasmid. We observed a significant increase in TUNEL-positive 661W cone cells in p70S6K1 shRNA treated groups relative to scramble controls at two different concentrations (Fig. 4b), indicating that S6K1 was critical for the survival of 661W cone cells. On the other hand, knockdown of either 4EBP1 or elF4E expression did not result in a higher number of TUNEL-positive 661W cone cells compared with scramble control groups (Fig. 4c), indicating an insignificant role of elF4E in 661W cone cell survival.

Moreover, we determined whether S6K1 was the downstream effector of PTEN neurotoxicity. Interestingly, we found that S6K1 knockdown induced 661W cone cell apoptosis to a similar degree as PTEN overexpression (Fig. 4d), indicating that S6K1 was possibly a major downstream mediator of mTOR. To confirm this possibility, we co-expressed PTEN and p70S6K1 in 661W cone cells and tested whether cone cell apoptosis induced by PTEN overexpression could be rescued by S6K1 expression. Indeed, we found that S6K1 significantly decreased the number of TUNEL-positive 661W cone cells in the presence of PTEN (Fig. 4d). These in vitro data confirmed that PTEN functions partly through S6K1 to regulate 661W cone cell apoptosis.

### S6K1 overexpression in cones enhances the survival of the cones in rd10 mice in vivo

**Fig. 5:**
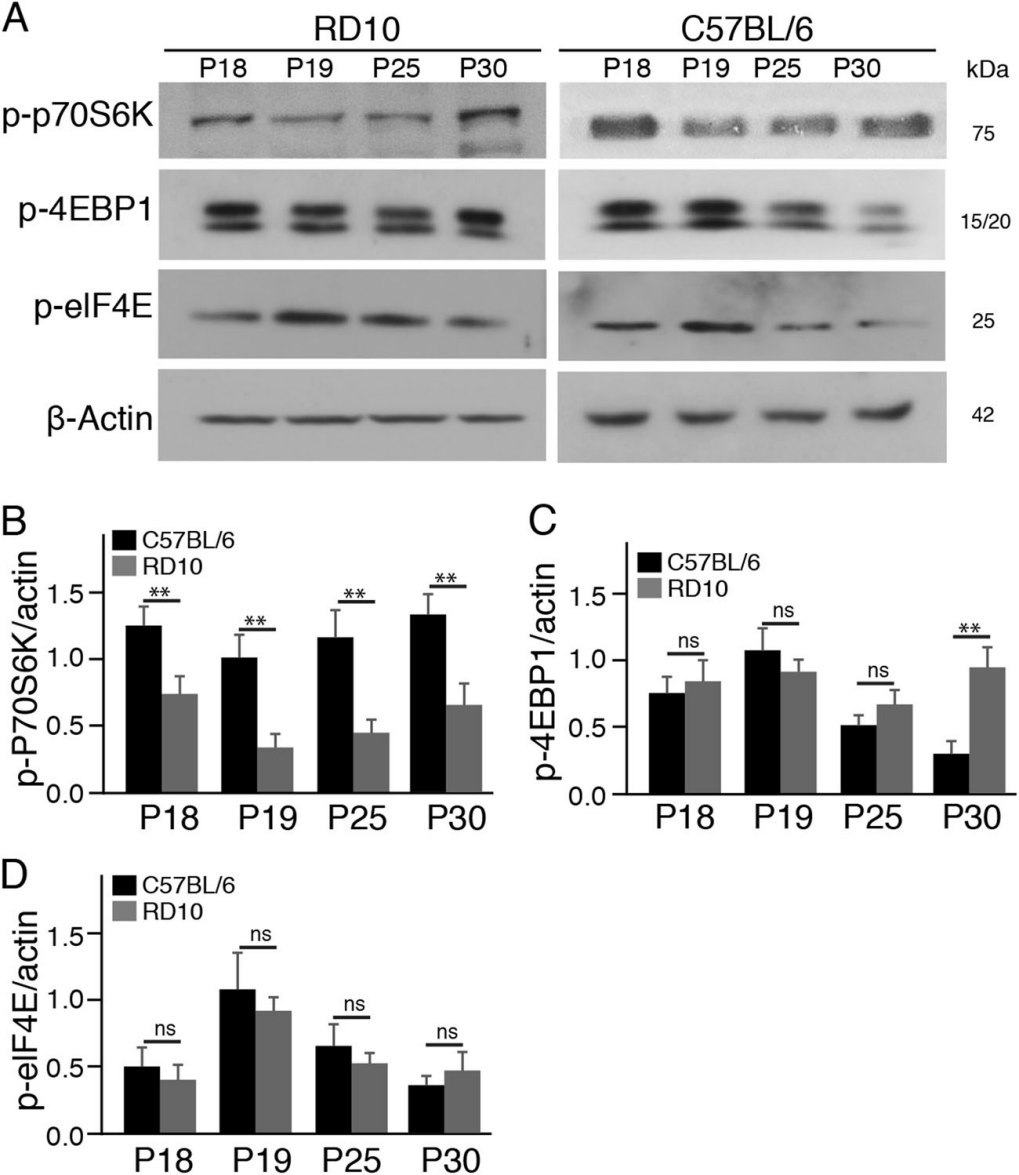
S6K1 is downregulated in the rd10 retina. **a** Western blots of two major downstream targets of mTOR, p-p70S6K, and p-4EBP1, in rd10 and age-matched C57BL/6 mouse retinas. β-actin levels were used as a loading control. **b–d** Densitometric analyses of the ratio of p-p70S6K (**b**), p-4EBP1 (**c**), and p-elF4E (**d**) to β-actin. Data are the mean±SD (*n* = 4). ns not significant, **p < 0.01

To verify whether S6K1 and 4EBP1 were differentially affected in the rd10 retina, we measured the protein expression levels of S6K1 and 4EBP1 in the rd10 retina at several time points. We found that the protein levels of p-p70S6K were decreased in rd10 retinas relative to C57BL/6J control retinas at all the time points (Fig. 5a, b). However, the protein levels of either p-4EBP1 or p-elF4E were comparable between rd10 and C57BL/6J control retinas (Fig. 5a, c, d). Our results demonstrated that S6K1 and 4EBP1, two immediate downstream effectors of mTOR, were differentially affected in the rd10 retina, and suggested that S6K1 was the potential downstream effector of mTOR in mediating rod and cone cell survival.

**Fig. 6:**
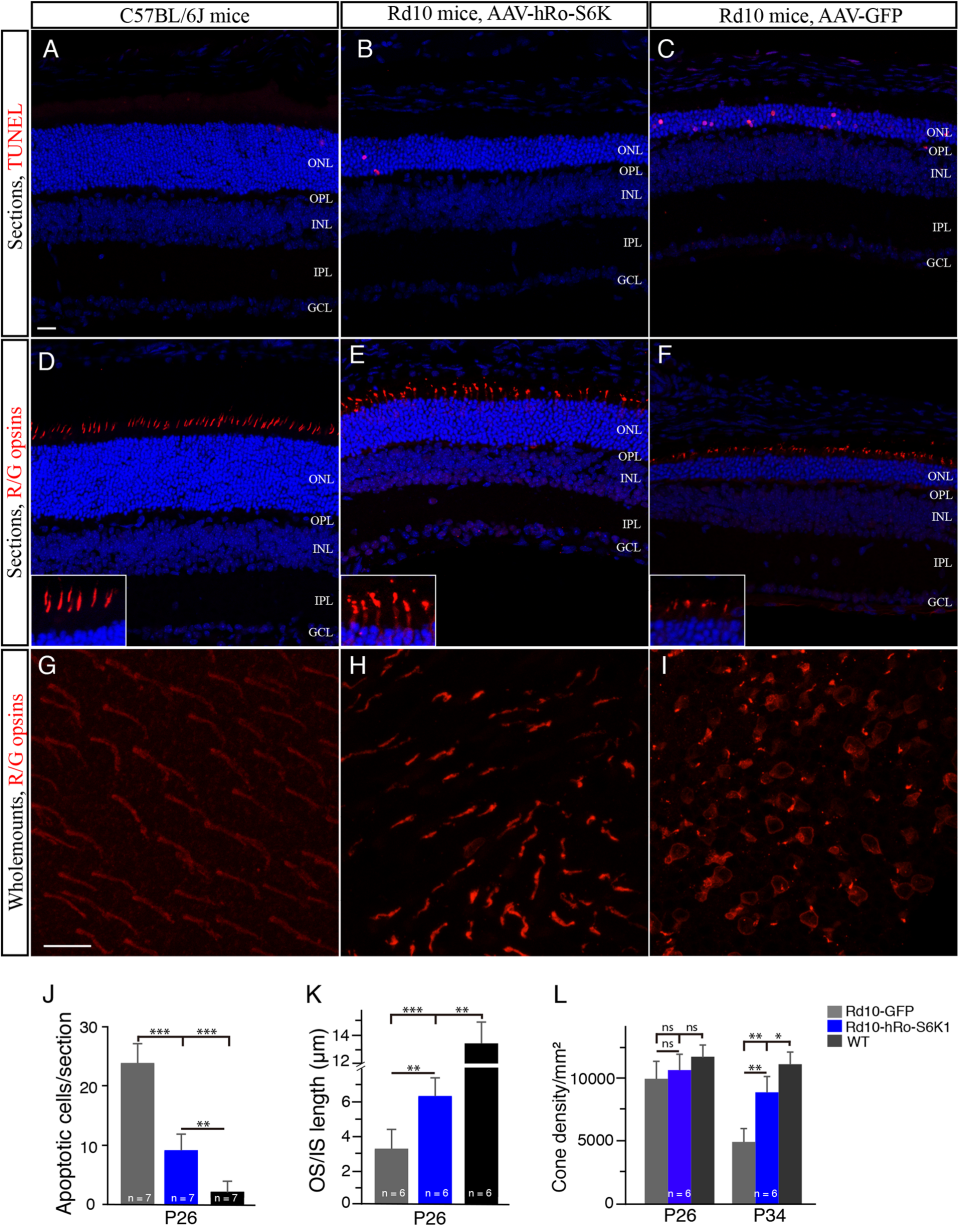
S6K1 overexpression in cones driven by a hRo promoter enhances the survival of the cones in rd10 mice. **a**–**c** Retinal sections from S6K1- and GFP-treated P26 rd10 mice and WT mice were stained with TUNEL (red). The nuclear layers in retinal sections were stained with diamidinophenylindole (DAPI, blue). **d**–**f** Retinal sections from S6K1- and GFP-treated P26 rd10 mice and WT mice were stained with an antibody against red/green opsin (red). The nuclear layers in retinal sections were stained with DAPI (blue). Insets are highly magnified images from respective images showing cone OS/ISs. ONL outer nuclear layer, OPL outer plexiform layer, INL inner nuclear layer, IPL inner plexiform layer, GCL ganglion cell layer, OS outer segment, IS inner segment. Scale bar: 20μm. **g**–**i** Confocal images of red/green cones in the dorsal retina of retinal flat mounts, 1mm superior to the center of the optic nerve are shown. Retinal flat mounts from P34 rd10 mice treated with S6K1 or GFP and age-matched WT mice were stained with an antibody against red/green opsins. Scale bar: 20μm. **j** Quantification of TUNEL-positive photoreceptors from retinal sections. **k** Plot of the length of cone OS/ISs measured on retinal sections. **l** Quantification of red/green cone density in the dorsal retina of retinal flat mounts. Results are presented as the mean±SD. ns, not significant, *p < 0.05, **p < 0.001, ***p < 0.0001

To confirm this possibility, we overexpressed S6K1 in cones in the rd10 retina using a AAV-S6K1 vector driven by a human red opsin promoter^15^. After a few weeks of inculation, we quantified apoptotic photoreceptor cells labeled by TUNEL in the outer nuclear layer (ONL). We found that the number of TUNEL-positive photoreceptor cells were significantly decreased in S6K1-treated P26 rd10 mice compared with GFP-treated rd10 controls (Fig. 6b, c, j), indicating that S6K1 treatment reduced apoptosis. Similarly, S6K1 treatment partially preserved cone outer and inner segments (OS/IS) in rd10 retinas (Fig. 6d–f). The measurement of cone OS/IS length on retinal vertical sections showed that cone OS/IS length was largely preserved in S6K1-treated P26 rd10 mouse retinas, when compared with age-matched rd10 controls (Fig. 6k). We then quantified the number of survival cones in flat-mounted retinas stained with an anti-red/green opsins antibody. At P26, average cone density was comparable between S6K1-treated rd10 mouse retinas and rd10 controls (Fig. 6l). By P34, however, we found that S6K1-treated rd10 mice appeared to have greater red/green cone density compared to GFP-treated rd10 mice (Fig. 6g–i). The average density of red/green cones in the dorsal retina of S6K1-treated rd10 mice was markedly higher compared with GFP-treated rd10 controls (by 74.5%; Fig. 6l). Similarly, the average density of blue cones in the ventral retina was significantly higher in S6K1-treated rd10 mice than in GFP-treated rd10 mice (data not shown). Therefore, S6K1 overexpression improved the survival of the cones in the rd10 retina. Meanwhile, we also observed that some rods were also saved in these experiments. We counted the number of nucleus rows labeled by DAPI (blue) in the ONL on retinal vertical sections for saving rods (Fig. 6b, c, e, f). We found that S6K1-treated P26 rd10 retinas contained 6 to 8 rows of photoreceptor nuclei, while 3 to 5 rows of photoreceptor nuclei were observed in P26 rd10 controls. Alternatively, we injected phRo-S6K1 construct into the subretinal space of newborn rd10 pups followed by electroporation. We found that in vivo electroporation produced similar results to the AAV-S6K1 vector showing that S6K1 overexpression in cone cells improved the cone cell survival (data not shown).

**Fig. 7:**
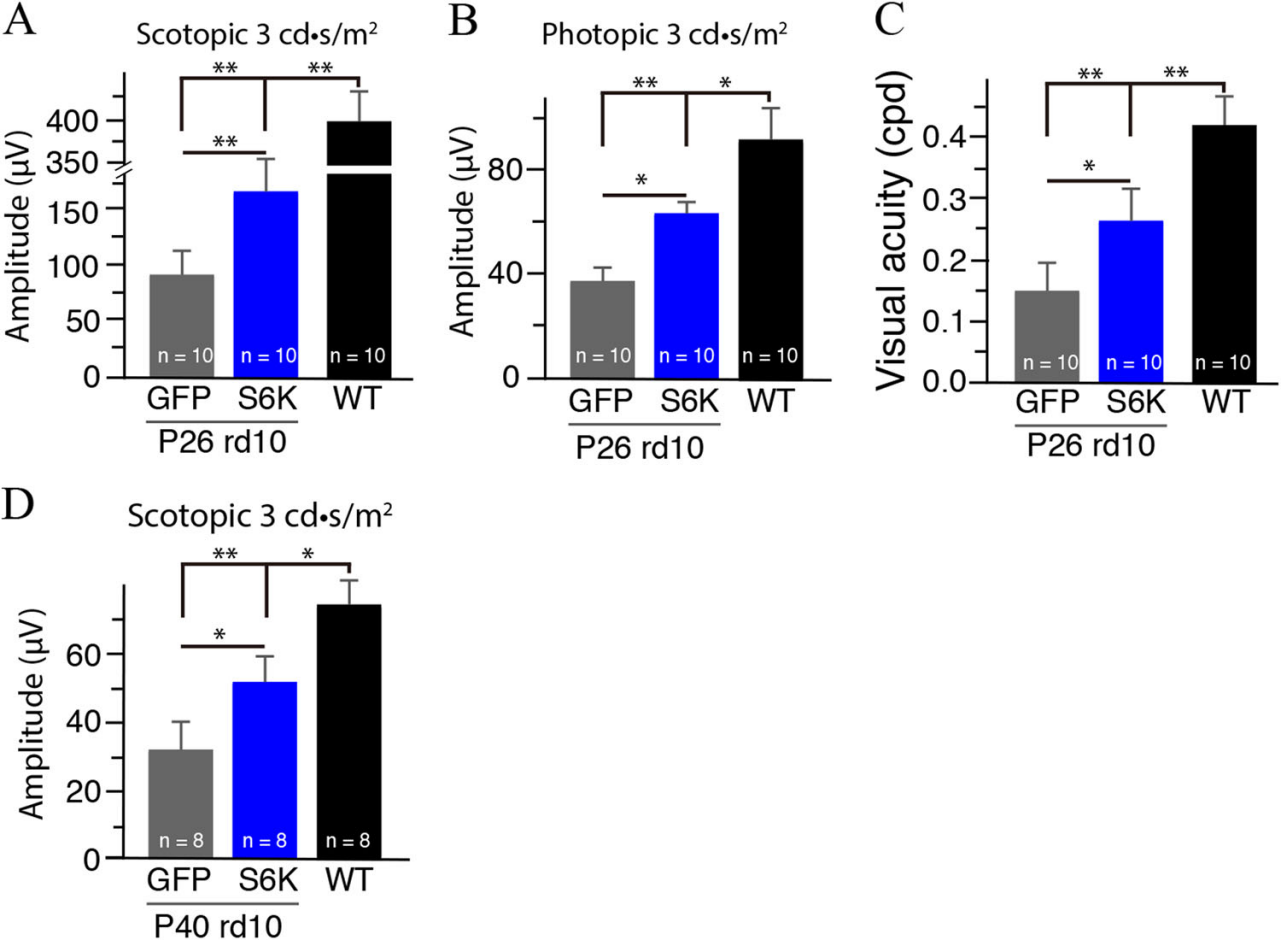
S6K1 treatment improved retinal function and visual performance in rd10 mice. **a** Average scotopic b-wave amplitudes from P26 S6K1- (blue bar) or GFP-treated rd10 mice (gray black bar). Age-matched C57BL/6 are shown as comparisons (black bar). **b** Average photopic b-wave amplitudes from the three groups of P26 mice. **c** Photopic visual acuity was measured by optokinetic responses in P26 S6K1- (blue bar) or GFP-treated rd10 mice (gray black bar) after overnight dark adaptation. **d** Average scotopic b-wave amplitudes from P40 S6K1- (blue bar) or GFP-treated rd10 mice (gray black bar). Results are presented as the mean±SD (*n* = 8–10). *p < 0.05, **p < 0.01

Moverover, we monitored retinal functional changes in the S6K1-treated rd10 mouse retina evaluated by ERG. We measured scotopic and photopic ERGs in P26 rd10 mice after S6K1 treatment. Markedly increased amplitudes of b-wave were observed in S6K1-treated rd10 mice under both scotopic and photopic conditions (Fig. 7a, b), indicating the preservation of photoreceptor function after S6K1 treatment. Meanwhile, visual performance was evaluated in S6K1-treated rd10 mice. Visual acuity was approximately 1.7-fold higher in P26 S6K1-treated rd10 mice than that in age-matched GFP-treated rd10 (*p* < 0.05; Fig. 7c), indicating improved visual acuity in S6K1-treated rd10 mice. Collectively, these data showed that S6K1 treatment improved retinal function and visual performance in rd10 mice. Interestingly, we observed that the rescue effect of S6K1 treatment was still present in P40 rd10 mice (Fig. 7d). Scotopic b-wave amplitudes were approximately 1.7-fold higher in S6K1-treated rd10 mice than in age-matched GFP-treated rd10 (*p* < 0.05; Fig. 7d). Therefore, functional preservation of photoreceptors by S6K1 expression could last for a long period of time.

### S6K1 overexpression in rods enhances the survival of the rods in rd10 mice in vivo

**Fig. 8:**
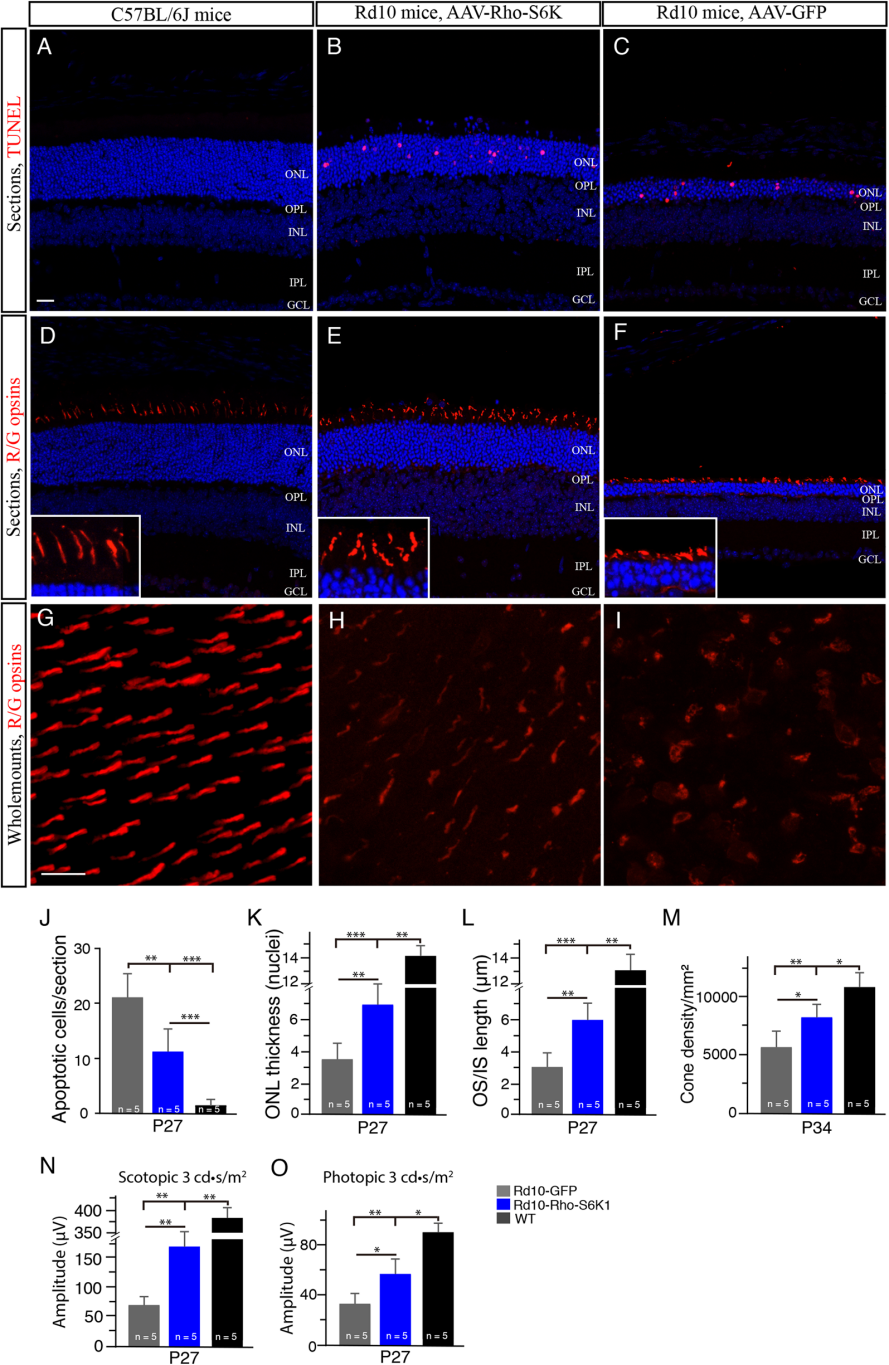
S6K1 overexpression in rods driven by a bovine rhodopsin promoter enhances the survival of the rods in rd10 mice. **a**–**c** Retinal sections from P27 rd10 mice treated with S6K1 or GFP and age-matched WT mice were stained with TUNEL (red). The nuclear layers were stained with DAPI (blue). **d**–**f** Retinal sections from P27 rd10 mice treated with S6K1 or GFP and age-matched WT mice were stained with an antibody against red/green opsin (red). The nuclear layers were stained with DAPI (blue). Insets are highly magnified images from respective images showing cone OS/ISs. ONL outer nuclear layer, OPL outer plexiform layer, INL inner nuclear layer, IPL inner plexiform layer, GCL ganglion cell layer, OS outer segment, IS inner segment. Scale bar: 20μm. **g**–**i** Confocal images of red/green cones in the dorsal retina of retinal flat mounts, 1mm superior to the center of the optic nerve are shown. Retinal flat mounts from P27 rd10 mice treated with S6K1 or GFP and age-matched WT mice were stained with an antibody against red/green opsins. Scale bar: 20μm. **j** Quantification of TUNEL-positive photoreceptors from retinal sections. **k** Plot of the ONL thickness from P27 rd10 mice measured in numbers of photoreceptor nuclei per column. **l** Plot of the length of cone OS/ISs measured on retinal sections. **m** Quantification of red/green cone density in the dorsal retina of retinal flat mounts. **n** Average scotopic b-wave amplitudes from P27 rd10 mice. Age-matched C57BL/6 are shown as comparisons (black bar). **o** Average photopic b-wave amplitudes from the three groups of P27 mice. Results are presented as the mean±SD. *p < 0.05, **p < 0.001, ***p < 0.0001

To examine whether S6K1 expression in rods could save the rods, we injected pRho-S6K1 construct containing a bovine rhodopsin promoter (Rho)^14^ into the subretinal space of newborn rd10 pups followed by electroporation. We found that the number of TUNEL-positive photoreceptor cells were decreased in S6K1-treated P27 rd10 mice compared with age-matched GFP-treated rd10 controls (Fig. 8b, c, j). In addition, we counted the rows of photoreceptor nuclei in the ONL on retinal vertical sections (Fig. 8b, c). Compared to rd10 controls, S6K1-treated P27 rd10 mice had a significantly thicker ONL (*p* < 0.01; Fig. 8k), indicating the reduction of rod cell death in S6K1-treated rd10 mice. Previous studies report that cone survival is coupled to rod survival^9,25^ Interestingly, we also observed morphological preservation of cones in rd10 mice (Fig. 8e, f). Qualification of the OS/IS length showed that cone OS/IS was partially preserved in S6K1-treated rd10 mice (Fig. 8l). Cone cell density between S6K1- and GFP-treated rd10 retinas was comparable at P27 (Fig. 8h, i). At P34, however, average cone density was significantly higher in S6K1-treated rd10 mice than GFP-treated rd10 mice (by approximately 47%; Fig. 8m). Higher amplitudes of b-wave were observed in the eyes of S6K1-treated rd10 mice compared with rd10 controls under both scotopic (Approximately 2.5-folds; Fig. 8n) and photopic conditions (Approximately 1.8-folds; Fig. 8o). Therefore, S6K1 overexpression in rods improved the rod survival and saved cones as well in rd10 mice.

## Discussion

In this study, we demonstrated that PTEN overexpression in 661W cone cells deactivated mTOR and its downstream target S6K1 activity and induced the 661W cone cell apoptosis in vitro. Moreover, we identified that S6K1 but not 4EBP1 was the downstream effector of PTEN neurotoxicity using gain- and loss-of-function approaches. We showed that S6K1 knockdown in 661W cone cells induced the 661W cone cell apoptosis to a similar degree as PTEN overexpression, whereas S6K1 overexpression improved the 661W cone cell survival in the presence of PTEN. Furthermore, our in vivo study supported the findings of our in vitro data. We found that PTEN upregulation in the rd10 mouse model of RP deactivated S6K1 activity. On the other hand, S6K1 overexpression either in rods or cones in rd10 mice promoted these photoreceptor survival and function and improved visual performance. Therefore, our in vitro and in vivo studies consistently demonstrated that S6K1 was the downstream effector of PTEN neurotoxicity and that S6K1 was critical for both rod and cone cell survival in the rd10 mouse retina.

The mTOR signaling pathway, which has key functions in regulating cell growth, survival and metabolism, is aberrantly activated in a number of neurological diseases^8^. The tumor-suppressor phosphatase PTEN negatively regulates the mTOR pathway^6,23^. Previous studies have shown that PTEN deletion up-regulate mTOR activity and promote axon regeneration in retinal ganglion cells and corticospinal neurons^13,26^. Similarly, the mTOR signaling pathway is very important for the maintenance of cone viability in the retina. Deactivation of the mTOR pathway is reported to accelerate cone death the rd1 mouse model of RP^10^. Consistent with these previous reports, our in vitro data from 661W cone cells confirmed that PTEN activity downregulated the mTOR pathway and induced 661W cone cell apoptosis, indicating the important role of the mTOR signaling in 661W cone cell survival. Moreover, we identified that S6K1 rather than 4EBP1 was critical for 661W cell survival, and that S6K1 was the downstream effector of PTEN neurotoxicity. Our in vitro data demonstrate that PTEN regulates 661W cone cell apoptosis partly through the inhibition of S6K1 activity. Furthermore, our in vivo data from rd10 mice confirmed the results of our in vitro studies. When we overexpressed S6K1 in rods using a rod-specific promotor, we found that S6K1 overexpression improved the rod survival in rd10 mice and eventually saved cones as well. These findings are consistent with previous reports that cone survival is coupled to rod survival^9,25^. In RP, however, some of cones survive for many months and even years after completion of the end phase of rod death suggests that cones are not totally dependent on adjacent rods for their survival^3,19^. Indeed, when we expressed S6K1 in cones using a cone-specific promoter, we found that S6K1 expression rescued the cone cells from degeneration in rd10 mice. To confirm the beneficial effect of S6K1 activity on cones, we co-expressed S6K1 with PTEN in 661W cone cells in vitro. We found that S6K1 expression rescued the 661W cone cell apoptosis induced by PTEN activity. Our results are strongly consistent with one previous report that mTORC1 activation in cones, which directly controls S6K1 activity, was sufficient to promote long-term cone survival in rd1 mice^10^. Meanwhile, we observed that some rods were also saved in these experiments. The reason for rod saving is unclear. One possibility is that non-specific expression of S6K1 in rods saved these rods, which in turn could help further improve the survival of cones. In this case, cones could possibly be saved directly by S6K1 overexpression in cones and indirectly by these saved rods. Collectively, our overall data confirmed that S6K1 is essential for the survival of both rods and cones in rd10 mice. Our results are consistent with previous observations that S6K1 plays a key role in the axon regeneration of retinal ganglion cells^13,27^, and that S6K1 activation promotes axon regeneration after the optic nerve crush^28^. Although our results have shown that PTEN signaling represents one of the upstream pathways that downregulates mTOR and S6K1 activity, the possibility of involvement of other pathways such as the serine-threonine kinase liver kinase B1 (LKB1)-AMP-activated protein kinase (AMPK) pathway, which acts directly on mTORC1 to regulate negatively mTOR activity^29^, should also be considered.

Taken together, our results indicated that inactivation of mTOR and S6K1 induced by PTEN activity is a possible mechanism underlying the progressive photoreceptor death in the rd10 retina. Conversely, PTEN deletion leads to reactivation of mTOR and S6K1, which allows rods and cones to regain some of cell survival viabilities characteristic of healthy photoreceptors. Similarly, S6K1 overexpression either in rods or cones in the rd10 retina rescued these photoreceptor cells from apoptosis induced by PTEN activity. Therefore, understanding the regulation mechanisms of mTOR and S6K1 activities during RP progression might lead to development of therapeutic approaches centered around S6K1 activation for treating patients who suffer from retinal degeneration.

## Materials and methods

### 661W cone cell culture

A mouse photoreceptor-derived cell line (661W cone cells) was generously provided by Dr. Muayyad Al-Ubaidi at University of Oklahoma^24^. The 661W cone cells express several markers of cone photoreceptors such as cone opsins, transducin, and cone arrestin^24^. 661W cells were seeded at a density of 5×10^4^ cells per well of 24-well plates and cultured in DMEM supplemented with 10% FBS, penicillin (100U/mL), and streptomycin (100mg/mL) at 37 °C in 95% air and 5% CO_2_. The 70% confluent 661W cells were transiently transfected with a mixture composed of lipofectamine 2000 (Invitrogen) and pCMV/Flag/WT-PTEN (200ng, Addgene), with overexpression of pCMV/Flag/dPDZ-PTEN (200 ng, Addgene) as the negative control. P70S6K1 overexpression was realized by transfecting pRK7-HA-S6K1-WT (Addgene), and pRK7-HA-S6K1-KR (Addgene) was used as the negative control.

DNA sequence for p70S6K1 shRNA was sourced from Sigma-Aldrich (St. Louis, Missouri). DNA oligos for sense and antisense sequences of p70S6K1 shRNA were synthesized from Integrated DNA Technologies, Inc. (Singapore), and annealed to form double-string structure by incubating for 4 min at 95 °C followed by 10-min incubation at 70 °C. The annealed oligo was ligated to pLKO.1-TRC vector at the site between endonuclease sites of *Age* I and *Eco*R I following protocol of T4 ligase (Invitrogen). Ligation mix was transformed into competent DH5 alpha cells, and positive colonies were selected and enlarge cultured for plasmid preparation. Plasmids of pLKO.1/p70S6K1-shRNA and the negative control vector pLKO.1-scramble shRNA were both extracted and purified using QIAprep Spin Miniprep Kit (QIAGEN).

To detect and quantify the number of apoptotic 661W cells, we labeled 661W cells by TUNEL staining, which labels partially degraded DNA. Nuclei were counterstained with the nuclear dye diamidinophenylindole (DAPI). Apoptotic cell counting was performed on three coverslips for each experiment. Cells were counted under a 40x objective in 10 adjacent fields along the diagonal axes of each coverslip. Results are expressed as the ratio of TUNEL-positive cells to DAPI-stained nuclei. These analyses were blindly conducted.

### Animals

Wild-type (C57BL/6J) mice, rd10 mice and PTEN^loxP/loxP^ mice were obtained from Jackson Laboratory (Bar Harbor, ME). Rd10 mice were backcrossed with PTEN^loxP/loxP^ mice, and the littermates from rd10/PTEN^loxP/loxP^ mice were used for experiments. The genotypes of mouse litters were determined by PCR and confirmed by Southern blot analysis of genomic DNA from tail biopsies. Animals were bred and maintained at the Centralized Animal Facilities (CAF) of The Hong Kong Polytechnic University on a 12-hour light–dark cycle with a room illumination of around 50lux and water and food ad libitum. All experimental procedures were approved by the Animal Subjects Ethics Sub-committee (ASESC) of The Hong Kong Polytechnic University and conducted in accordance with the ARVO statement for the use of animals.

### AAV injection

The plasmid CAG-Cre was purchased from Addgene (#13775). The plasmid hRo-Cre was constructed by replacing CAG with hRo (human red opsin promoter)^15^, a generous gift from Dr. Jeremy Nathans at Johns Hopkins University. The constructs hRo-Cre and hRo-S6K1 were packaged into AAV2/8 serotype virus by the University of Pennsylvania Vector Core. We injected subretinally an AAV vector (~0.5 μL) into one eye of mice around postnatal day 5 (P5) with a 32-gauge blunt-ended needle as previously described by us^30^. In controls, mice received a single subretinal injection of AAV-GFP.

### In vivo electroporation

For construction of pRho (bovine rhodopsin promoter)-S6K1, the promoter region of phRo-S6K1 was replaced by Rho, which was purchase from Addgene (#13779)^14^. Subretinal injection and in vivo electroporation of the mouse retina were performed as previously described^14^. Newborn mouse pups (P0 or P1) were anesthetized by chilling on ice, and a small incision is made in the eyelid and sclera with a 30-gauge needle. pRho-S6K1 or phRo-S6K1 plasmid (0.5μL at 5μg/μL in PBS) was injected into the subretinal space of newborn mouse pups using a Hamilton syringe with a 32-gauge blunt-ended needle under a dissecting microscope. After DNA injection, 80 V pulses were applied using square pulse electroporator ECM830 (BTX, Japan).

### Immunocytochemistry and confocal imaging

The mice were aged up to P40 after AAV injection. Animals were sacrificed with an overdose of sodium pentobarbital. Eyes were quickly enucleated after a reference point was made to label the superior pole and the retinas were dissected free of vitreous and sclera in carboxygenated Ames' Medium (Sigma-Aldrich, St. Louis, MO), and then fixed in 4% paraformaldehyde (PFA) in 0.1M phosphate buffer (PB), pH 7.4 for 0.5–1 h. Some retinas were sectioned serially at a thickness of 10–12μm using a cryostat. Both whole-mounted retinas and cross sections were blocked in a solution containing 3% normal goat serum (NGS), 1% bovine serum albumin (BSA), and 0.3% Triton X-100 in PBS (pH 7.4) for 1 h. Primary antibodies used were rabbit anti-red/green opsin (1:500, Chemicon, Temecula, CA), rabbit anti-blue opsin (1:500, Chemicon, Temecula, CA), and rabbit anti-Cre recombinase (1:500, Covance, San Diego, CA).

The primary antibodies were diluted with a blocking solution (1% NGS, 1% BSA, 0.1% Triton X-100 in PBS) and applied to sections or whole-mounted retinas from overnight to 3 days at 4 °C. After blocking and rinsing, a secondary antibody conjugated to either Alexa 488 (1:500; Invitrogen) or Alexa 594 (1:500; Invitrogen) was applied to sections or whole-mounted retinas for 2 h at room temperature. Sections and whole-mounted retinas were rinsed, and cover slipped in Vectashield mounting medium (Vector Laboratories, Burlingame, CA).

Confocal micrographs of fluorescent specimens from retinal flat-mounted preparations and vertical sections were captured using a Zeiss LSM 800 confocal laser scanning microscope with Airyscan module (Carl Zeiss, Oberkochen, Germany) equipped with argon and helium-neon lasers. Plan-Apochromat 63x/1.4 or 40x/1.4 oil immersion objectives were used. Image scale was calibrated, and if necessary, brightness and contrast were adjusted using Photoshop CS8 software (Adobe Systems, San Jose, CA).

### Electroretinographic (ERG) analysis

ERG was recorded by using the electrophysiological RETI-animal (Roland Consult, Brandenburg, Germany) system as described by us^20,22^. Briefly, mice were adapted to darkness for 12h. All of the following procedures were performed under deep red illumination. The animals were anaesthetized intraperitoneally with a mixture of Dormitor (1mg/kg medetomidine hydrochloride; Pfizer, UK) and Ketamine. The mice were then placed on a heated platform to keep their body temperature constant (37±0.5 °C) during the measurements. Mouse pupils were dilated using a single drop of 1% Mydriacyl (Alcon, Ontario, Canada). Flash ERG was measured using a gold wire corneal electrode, a forehead reference electrode, and a ground electrode near the tail. A scotopic ERG was obtained from dark-adapted animals at the following increasing light intensities: 0.01 and 3cd-s/m^2^. A photopic ERG was recorded following 10-minute light adaptation on the background light intensity of 3cd-s/m^2^. ERG b-wave was measured from the trough of the a-wave to the peak of the positive wave or, when the a-wave was not present, from baseline to the peak of the first positive wave.

### Optokinetic tracking

Optokinetic tracking was performed using a virtual optokinetic system (OptoMotry, CerebralMechanics, Canada) as previously described by us^20,22^. This test measures the tendency of an animal to follow with the head the movements of the surrounding environment, and is a good predictor of visual acuity. In practice, mice stood on an elevated platform in the epicenter of an arena surrounded by computer monitors, and a camera images the behavior of the animal from above. A cylinder comprised of a sine wave grating is drawn in three-dimensional coordinate space and rotates around the animal. Animals track the grating with reflexive head and neck movements. Spatial frequency thresholds can be measured by systematically increasing the spatial frequency of the grating at 100% contrast until animals no longer track.

### Western blot analysis

Cell lysates of mouse retinas or 661W cells were prepared in RIPA buffer (Sigma-Aldrich) with phosphatase inhibitor cocktails (Sigma-Aldrich). Cell debris was removed by centrifugation at 10,000×*g* at 4 °C for 10 min and protein concentration of the cell lysate was determined using a Bio-Rad Protein Assay Kit. The cell lysate was resolved in a 7.5% gel by sodium dodecyl sulfate polyacrylamide gel electrophoresis (Bio-rad) and trans-blotted onto a PVDF membrane (Millipore) at 100mA for 2 h. The membrane was then blocked with 5% non-fat dried milk in TBST buffer (10mM Tris, 150mM NaCl, 0.05% Tween20, pH 8.0) and incubated overnight at 4 °C with the antibodies (Cell Signaling Technology, MA) against (i) the phosphorylated form of PTEN (Ser380/Thr382/383) (1:2000), Akt (Ser473) (1:2000), mTOR (Ser2448) (1:1000), and p70S6K (Thr412) (1:1000), respectively, and (ii) total proteins of PTEN (1:2000), Akt (1:2000), and mTOR (1:1000), respectively. On the following day, HRP-conjugated anti-rabbit IgG (1:2000, Cell Signaling Technology, MA) was added and the HRP signal was detected using WesternBright ECL (Advansta Inc., Menlo Park, CA). The ratio of the optical density of the protein product to the internal control (β-actin, cat no. 3700, Cell Signaling) was obtained and was expressed as ratio or percentage of the control value in the Figures. The specificity of all the antibodies used for western in mouse has been validated by the manufacturers.

### Data analysis

For length measurement of cone outer and inner segments, only sections passing through the optic nerve head were analyzed. Three sections per retina were examined and measurements taken 1mm from the optic nerve on both sides. Quantification of surviving cones stained with red/green opsins and blue opsin was conducted in retinal whole-mounts. Sampling areas were two 240×240μm squares along the dorsal-ventral axis of retinal whole-mounts per retina, 1mm from the optic nerve on both sides. The raw counts were then converted into cells/millimeter^2^.

### Statistical analysis

All data were expressed as means±SDs. ANOVAs with Bonferroni’s and Dunnett’s post hoc tests for multiple comparisons were performed with Origin (OriginLab) and programs written in MATLAB (Mathworks) on full data sets to detect significant differences in the means. A *p*-value < 0.05 was considered statistically significant.
